# Influence of compliance to antithrombotic agents on perioperative morbidity and mortality

**DOI:** 10.1186/s44158-023-00123-5

**Published:** 2023-10-18

**Authors:** Olivier Duranteau, Ayoub Hamriti, Brigitte Ickx, Turgay Tuna

**Affiliations:** 1 Anesthesiology Service, CUB-ULB Hôpital Erasme, Brussels, Belgium; 2 Intensive Care Unit, HIA Percy, Clamart, France

**Keywords:** Antiplatelets agents, Anticoagulation, Peri-operative medicine, Personal prescription, Bridge

## Abstract

For patients on antithrombotic medication, the outcome of a planned surgery depends on the adjustment of their daily treatment. This study aimed to assess the impact of non-compliance to published recommendations about antithrombotic agents, specifically those provided by the Groupement d'Intérêt pour l'hémostase Peri-opératoire (GIHP), on patient morbidity and mortality.

A prospective cohort observational monocentric study was conducted over a 7-month period in 2019 in an academic hospital. The study included patients on antithrombotic agents scheduled for elective surgery, excluding cardiac surgery. The primary endpoints were morbidity and mortality at 1 month according to GIHP guidelines compliance. Blood loss during surgery and length of hospital stay were secondary endpoints.

Among the 589 patients included in the study, 87 complications were recorded, resulting in a morbidity rate of 14.8%. Thirty-six patients experienced hemorrhagic events, leading to 8 deaths, while 33 patients suffered from ischemic events, resulting in 3 deaths. Additionally, 18 patients died from causes unrelated to hemorrhage or ischemia. Compliance with GIHP guidelines was observed in 62% of patients. Statistical analysis did not reveal a significant association between adherence to guidelines and morbidity (*p* value = 0.923), nor between adherence and 1-month survival (*p* value = 0.698). Similarly, no statistically significant relationships were found between compliance and intraoperative bleeding volume (*p* value = 0.087), postoperative bleeding (*p* value = 0.460), or length of hospital stay (*p* value = 0.339).

This study did not identify any substantial associations between non-strict adherence to GIHP recommendations and patient outcomes, including morbidity, mortality, and bleeding. However, it is important to interpret these findings with caution, considering the study’s limitations and the need for further research in this area.

**Supplementary Information:**

The online version contains supplementary material available at 10.1186/s44158-023-00123-5.

## Introduction

The evolution of anticoagulant and antiplatelet therapies has markedly improved the mitigation of complications linked with chronic cardiovascular disorders. These therapies minimize the incidence of ischemic and thromboembolic events, albeit with an increased risk of bleeding upon too short cessation [1]. In the setting of planned surgery, too short a cessation of these medications may increase the risk of bleeding. Alternatively, excessive stopping time may induce thrombotic episodes. Therefore, achieving an intricate equilibrium between these considerable risk categories is important [2, 3].

In the context of impending surgeries, specific patients require modifications in their routine medication protocol. The dilemma of whether to suspend antithrombotic medication arises for some individuals. The therapeutic plan encompasses several aspects, divisible into three main domains: treatment identification, treatment indication (such as primary prevention or mechanical aortic valve), and the planned surgical procedure type [4]. Depending on these parameters, antithrombotics may be continued, halted, or substituted with alternate drugs [5].

Several elements account for this process, ranging from the transmission of guidelines to the adherence of physicians, the patient’s observance of medical prescriptions, and the understanding of these recommendations by physicians and patients [6, 7].

Additionally, certain physicians, despite having a thorough understanding of the guidelines, opt not to comply with them, choosing to follow local practices diverging from the guidelines. Although this behavior is noted across medical centers, its influence on patient care has yet to be adequately assessed [8, 9].

Numerous medical societies have scrutinized this issue, with some formulating recommendations aimed at improving compliance [10–13]. However, limited research and international guidelines have detailed the proper execution of these recommendations and their impact on patient outcomes.

This study primarily aimed to assess the consequences of non-adherence to these guidelines on patient morbidity (occurrence of either hemorrhagic or ischemic events), mortality, and hospital stay duration.

## Materials and methods

### Study design

A prospective, cohort, observational, single-center study was undertaken at an academic hospital over a 7-month duration, from 1 January to 31 July 2019, adhering to the STROBE recommendations (Supplemental Table 1).

### Study population

The study encompassed all patients lined up for elective surgery under general anesthesia at the main operating theatre of Erasme Hospital, Belgium. Patients undergoing urological, pulmonary, and gastrointestinal endoscopy were excluded due to organizational factors. Cardiac surgery patients were exempted owing to the inherent bleeding risk involved in this surgery type. Patients participating in other interventional trials were also excluded. Pregnant women, children, and adults incapable of giving informed consent were further excluded. The attending physician determined the specific anesthetic protocol, with guidelines encompassing target mean arterial pressure (mean arterial pressure greater than 65 mmHg or a 30% reduction if hypertensive) and anesthesia depth parameters (bispectral index target of between 40 and 60). The attending anesthetist had the liberty to choose anesthetic agents, administer fluids, and utilize catecholamines. Tranexamic acid was given when the intraoperative bleeding volume was above 500 mL. The surgeon's experience was not accounted for.

### Ethics

Ethical approval for the study was obtained from the Ethics Committee of Erasme Hospital, Anderlecht, Belgium (Ethics Committee N°P2018-504) on 19 October 2018. Written consent was obtained from all participating patients.

### Data collection protocol

The patient screening took place one week before the surgery. Pre-operative data were collected before the instigation of general anesthesia during the safety checklist. The surgeon in charge noted the peri-operative data at the surgery’s close. The principal investigator extracted the post-operative data, including 30-day outcomes, from the patient's medical files.

### Endpoints

The primary outcome was a combined measure of mortality and morbidity within 30 days, characterized by the occurrence of either hemorrhagic or ischemic events. A hemorrhagic event was defined as any situation necessitating a surgical procedure, irrespective of its complexity, to manage a surgical complication. An ischemic event was defined as the necessity of a new surgical procedure, an endovascular procedure, or the necessity of a high dosage of antithrombotic agents. The study also assessed adherence to French recommendations (described in the “Recommendations” section) and its correlation with other outcomes. Secondary outcomes included intraoperative surgical blood loss (following ESAIC guidelines [14]), duration of hospital stay, and rates of reoperation for any kind.

### Recommendations

The guidelines for this study were adopted from the French language recommendations provided by the Interest Group for Perioperative Haemostasis (GIHP). Registrars conducted each anesthesia consultation, with complex cases being reviewed by a senior consultant. In complex scenarios, senior doctors could consult with two internal referral physicians for advice.

These GIHP recommendations were presented in a department-wide meeting at the start of each academic year, and a printed version, illustrating various cases and protocols, was given to the participants. No formal tests or regular knowledge assessments were performed post-presentation.

To the best of our knowledge, the patients’ surgeons and general practitioners did not receive any information about the GIHP’s recommendations and their knowledge could therefore not be certain.

### Database

Data collected for analysis encompassed demographic information (such as age, weight, height, smoking status, pre-existing diabetes, type of surgery), treatment specifics (treatment indication, specific antithrombotic medication, requested cessation date by the anesthetist, actual cessation date, adherence to Francophone recommendations, reasons for non-compliance), preoperative data (preoperative activated partial thromboplastin time, prothrombin time, international normalized ratio, hemoglobin level, platelet count, glomerular filtration rate), operative data (bleeding volume), and postoperative data (postoperative bleeding, duration of hospital stay, number of reoperations, patient status at 1 month). Due to unavailability, the surgery duration was not recorded for all patients, thus limiting its incorporation into our study despite its potential relevance. Patients were classified as non-compliant from the day their actual medication intake deviated from the guidelines prior to surgery. The study did not differentiate between instances where medication was taken too close to or too far ahead of the surgery. As for the cessation date, the anesthetist could either provide an exact cessation date or use phrases like “last taken on D-xx” or “no discontinuation” in the treatment plan.

### Statistics

#### Tests

Continuous variables were expressed as mean ± standard deviation (SD) or median along with the range. Categorical values were presented as absolute numbers and percentages. All statistical tests were two-tailed, with a *p* value less than 0.05 considered statistically significant. Data analysis was executed using IBM SPSS 28 for Mac OS X (IBM Corporation, Somers, NY) or R, version 3.4.1 (R Programming). The study employed Analysis of variance (ANOVA) using the “aov” function from the R Studio "mosaic" package, while Chi-square tests were performed using the “chisq.test” function from the R Studio "stats" package.

#### Sample size calculation

The study's sample size was set at 600 patients, based on previous research indicating a non-compliance rate of approximately 20% in academic centers and a postoperative complication risk of around 10% regarding ischemic or bleeding events. The chosen sample size aimed to achieve 80% power with a significance level of 5%. An additional 60 patients (10%) were added to account for potential errors in data collection or loss of follow-up.

## Results

**Fig. 1:**
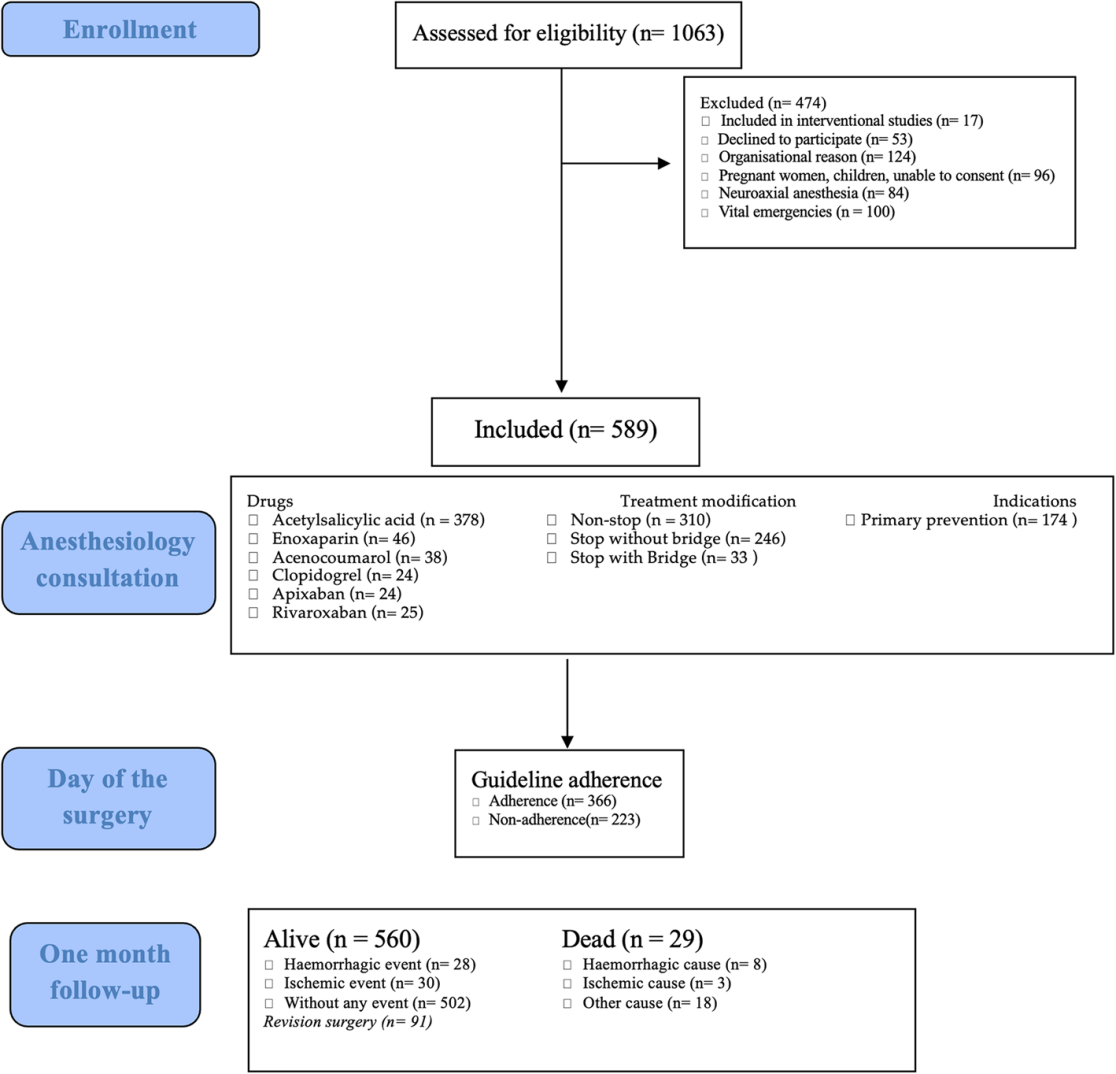
Consort flow diagram

In 2019, the Erasme Hospital operating theatre performed a total of 7850 operations, with 1063 patients receiving treatment involving at least one antithrombotic drug. From this pool of eligible patients, 589 individuals were selected for inclusion in the study, excluding 90 patients undergoing cardiac surgery. The study spanned from January 1st to July 31st, 2019, as depicted in the flowchart Fig. 1. We did not reach the planned 600 patients because of a problem in counting the number of patients included, which resulted in several inclusion numbers being skipped and the study being stopped prematurely.

### *Patient characteristics *(Fig. 1)

The selected cohort consisted of 589 patients with an average age of 65 years. The cohort's average weight and height were 79.9 kg and 169.5 cm, respectively, yielding a mean body mass index of 28.75 kg/m^2^. Females constituted 40.5% (*n* = 239) of the cohort, while 30.6% (*n* = 180) had a history of diabetes, and 24.4% (*n* = 144) were identified as smokers, as tabulated in Table 1. It was observed that 52.6% (*n* = 310) of the patients did not require discontinuation of treatment following the recommendations. When discontinuation was necessary, anesthetists predominantly used an exact date format (24.6%, *n* = 76), with only 7.2% of cases (*n* = 22) adopting the “last taken on D-xxx” terminology.

**Table 1:** Demographic and preoperative biologic data (*n* = 589)

	Guideline’s adherence group		Guidelines non-adherence group		*P* value
	Mean	SD	Mean	SD	
BMI (kg/m^2^)	27.36	5.96	29.69	32.91	0.13
Weight (kg)	79.11	18.87	78.03	17.77	0.25
Height (cm)	169.50	9.73	167.63	12.85	0.99
Age (years)	78.06	152.30	81.33	144.85	0.02*
APTT (Sec)	25.73	5.64	25.67	9.87	0.64
PT (%)	89.50	26.51	91.98	25.01	0.23
INR	1.23	1.55	1.10	0.31	0.05
Hb (g/dL)	12.69	2.42	12.53	2.30	0.68
Platelets (/mm^3^)	311 567.68	94 745.81	251 420.48	112 501.76	0.18
GFR (mL/min/1.73 m^2^)	78.14	29.41	68.78	31.09	0.15

*BMI* Body mass index, *APTT* Pre-operative activated partial thromboplastin time, *PT* Pre-operative prothrombin time, *INR* Pre-operative international normalized ratio, *Hb* Hemoglobin, *GFR* Glomerular filtration rate

The study incorporated both scheduled and relative emergency surgeries (ie that could be delayed to respect the GIHP guidelines). 9.4% (n = 100) of the patients included were in life-threatening emergency situations and were therefore unable to stop their treatment. These patients were therefore not included in the study and thereby not included in the analysis. In about one-third of the cases (33.0%, *n* = 194), the urgency was relative, allowing the surgical procedure to be planned according to the GIHP guidelines.

Surgical indications predominantly consisted of gastrointestinal procedures (28%, 163 cases), followed by orthopedic (20%, 121 cases), and vascular surgeries (17%, 99 cases), as demonstrated in Table 2. Of the total cohort, 62.1% (*n* = 366) adhered to the GIHP recommendations.

**Table 2:** Surgical specialty repartition regarding adherence to guidelines

	Adherence to guidelines				Dead	Alive with consequences	Alive without consequences	Total	
								(Amongst specialities)	
	No		Yes						
	*N*	*%*	*N*	*%*	*N*	*N*	*N*	*N*	*%*
Total	223	100.00%	366	100.00%	29	58	502	589	100.00%
No identification	0	0.00%	7	1.90%	0	0	7	7	1.20%
Anesthesia	0	0.00%	2	0.50%	0	0	2	2	0.30%
Digestive	64	28.70%	99	27.00%	11	13	139	163	27.70%
Gynecology	5	2.20%	11	3.00%	2	2	12	16	2.70%
Neurosurgery	13	5.80%	54	14.80%	1	10	56	67	11.40%
Obstetrics	0	0.00%	4	1.10%	0	0	4	4	0.70%
Ear-nose-throat	2	0.90%	1	0.30%	0	0	3	3	0.50%
Orthopedics	40	17.90%	81	22.10%	4	8	109	121	20.50%
Plastic surgery	9	4.00%	11	3.00%	2	2	16	20	3.40%
Thoracic	24	10.80%	14	3.80%	1	5	32	38	6.50%
Transplantation	8	3.60%	13	3.60%	0	3	18	21	3.60%
Urology	12	5.40%	16	4.40%	0	1	27	28	4.80%
Vascular	46	20.60%	53	14.50%	8	14	77	99	16.80%

Five patients could not comply with the anaesthetist’s instructions due to misunderstanding, rendering analysis of these instances unfeasible The primary causes of non-adherence were incorrect indications provided during consultation by the anaesthetist (76%, *n* = 170), followed by incorrect indications by the responsible surgeon (20%, *n* = 45), and incorrect instructions by the patient's general practitioner in 9 cases (4%). Five patients could not comply with the anesthetist’s instructions due to misunderstanding, rendering analysis of these instances unfeasible.

Acetylsalicylic acid (80 mg dose) emerged as the most prevalent drug during preoperative consultations, administered to 64.1% (*n* = 378) of the patients. Table 3 illustrates the distribution of other agents used. Primary prevention was the main indication for most patients (*n* = 174), equating to 29.5% of the total cohort or 46% of the ASA prescriptions.

**Table 3:** Antithrombotic medication prescription distribution

	Adherence to guidelines				Justification		Total	
	No		Yes		Sclerosis of arterial system and left heart	Venous system and right heart		
	*N*	%	*N*	%			*N*	%
Total	223	100.00%	366	100.00%	356	233	589	100.00%
No treatment	0	0.00%	26	7.10%	0	26	26	4.40%
Acenocoumarol	18	8.10%	20	5.50%	35	3	38	6.50%
Acetylsalicylic acid	150	67.30%	228	62.30%	186	192	378	64.20%
Acetylsalicylic acid + dipyridamole	0	0.00%	1	0.30%	0	1	1	0.20%
Apixaban	11	4.90%	13	3.60%	24	0	24	4.10%
Clopidogrel	11	4.90%	13	3.60%	24	0	24	4.10%
Dabigatran	3	1.30%	5	1.40%	8	0	8	1.40%
Edoxaban	1	0.40%	3	0.80%	4	0	4	0.70%
Enoxaparin	13	5.80%	33	9.00%	36	10	46	7.80%
Unfractionated heparin	1	0.40%	3	0.80%	4	0	4	0.70%
Nadroparin	1	0.40%	1	0.30%	2	0	2	0.30%
Rivaroxaban	8	3.60%	17	4.60%	25	0	25	4.20%
Tinzaparin	6	2.70%	3	0.80%	8	1	9	1.50%

*Sclerosis of the arterial system and left heart*: Cerebral or coronary infarction, carotid atheroma, venous system, and right heart: Deep vein thrombosis or pulmonary embolism

*No treatment*: The patient was supposed to take a treatment a long time before the surgery but has stopped on his own a long time before also. The patient’s medical file stated that the patient should take aspirin as primary prevention and the patient chose of his own accord to stop the treatment long before surgery. In this case, there was no change in the patient not taking the treatment

### Primary endpoint

Of the 589 included patients, 87 reached the predefined combined primary endpoint, yielding a morbidity rate of 14.8%, and a mortality rate of 5%. This included 36 patients who encountered bleeding events (resulting in 8 fatalities) and 33 patients who endured ischemic events (resulting in 3 fatalities). An additional 18 deaths occurred due to reasons unrelated to bleeding or ischemia, as displayed in Table 4. Additionally, 91 patients (15.4%) necessitated reoperation. In the case of ischemic events, prolonged discontinuation of antiplatelet therapy was the primary cause in most instances (*n* = 28 out of 33), despite guidelines suggesting a short delay surrounding the surgical procedure to attain a therapeutic safety window. Bleeding events primarily occurred in two contexts: persistent antiplatelet therapy during high-risk procedures (*n* = 17 out of 36) and insufficient understanding of the cessation guidelines for direct oral anticoagulants (DOACs) (*n* = 19 out of 36), particularly in patients with renal insufficiency. No statistically significant association was found between adherence to GIHP guidelines and morbidity (*p* value = 0.923) or between adherence to GIHP guidelines and 1-month survival (*p* value = 0.698).

**Table 4:** Adherence to guidelines and consequences on mortality or morbidity

	Adherence to guidelines		Total	
	No	Yes		
Dead (within 30 days)	12	17	29	5%
Alive with consequences	22	36	58	10%
Alive without consequences	189	313	502	85%
Total	223	366	589	
	37.90%	62.10%	100%	

Adherence to the guidelines was weighted against the confounding factors of morbidity and mortality (weight, BMI, type of surgery, age, sex, duration of surgery) and no difference was found compared with the general results.

### Secondary endpoints

**Fig. 2:**
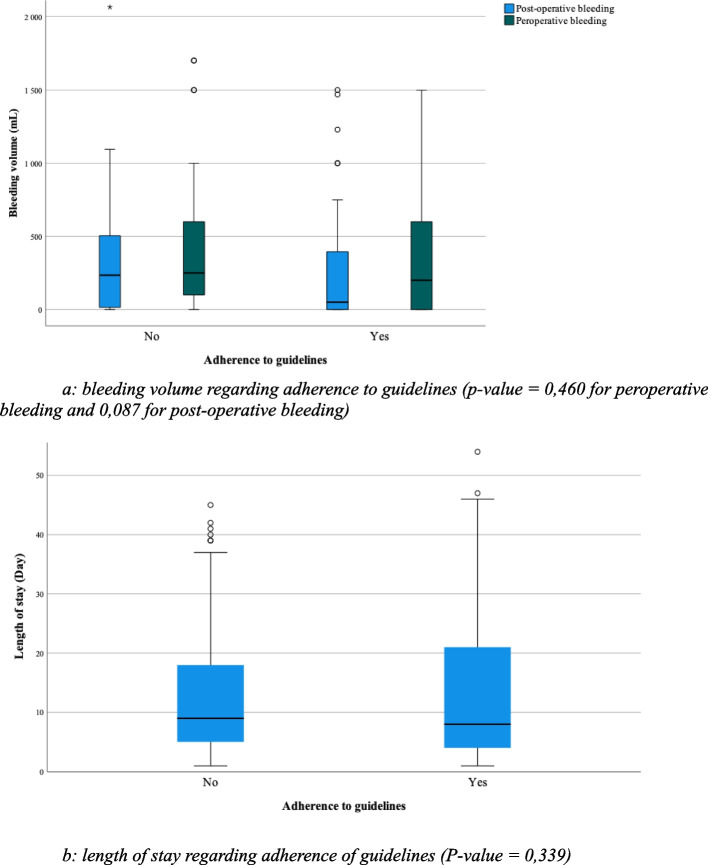
Bleeding volume and length of stay regarding adherence to guidelines. **a** Bleeding volume regarding adherence to guidelines (*p* value = 0.460 for perioperative bleeding and 0.087 for postoperative bleeding). **b** Length of stay regarding adherence to guidelines (*P* value = 0.339)

The average intraoperative (surgical) and postoperative bleeding volumes were recorded as 442 mL and 258 mL, respectively, with an average length of hospital stay of 17 days (Fig. 2). No significant correlation was observed between adherence to recommendations and intraoperative (*p* value = 0.087) or postoperative bleeding volume (*p* value = 0.460). Furthermore, no significant variation was seen in the length of hospital stay (*p* value = 0.339).

Five patients could not comply with the anesthetist’s instructions due to misunderstanding, rendering analysis of these instances unfeasible. The primary causes of non-adherence were incorrect indications provided during a consultation by the anesthetist (76%, *n* = 170), followed by incorrect indications by the responsible surgeon (20%, *n* = 45), and incorrect instructions by the patient's general practitioner in 9 cases (4%).

## Discussion

To the best of our knowledge, this investigation pioneers the exploration of the relationship between adherence and non-adherence to antithrombotic therapy guidelines and its ramifications on patient morbidity and mortality. Intriguingly, our analysis revealed no substantial differences in morbidity or mortality between patients adhering to and not adhering to the guidelines. Misdirection during preoperative consultations by the anesthetist surfaced as the principal source of non-compliance.

Our observational study identified non-compliance with the GIHP recommendations at a rate of 37.1%, which aligns with averages cited in various investigations concerning compliance with scholarly guidelines [15]. Existing literature underscores that compliance rates with such guidelines are generally modest, with strict adherence oscillating between 30 and 70% across different medical domains [16–19] and about 80% compliance with medication prescribing recommendations. The key factors contributing to this discrepancy include unique clinical scenarios that remain ambiguously defined in the guidelines, coupled with variations in local practices and historical habits.

The practical utility of promulgating an extensive set of guidelines with such limited adherence rates necessitates reconsideration. Similarly, it evokes the question of ongoing education pertaining to the application of these guidelines. The deployment of monitoring instruments to ensure the quality of knowledge dissemination and prescription accuracy could be beneficial.

Efforts to improve practice should focus on fortifying the understanding of anesthetists regarding these guidelines. The plethora of guidelines disseminated by disparate scientific societies may engender confusion, and the establishment of lucid local protocols has not significantly boosted adherence to these guidelines. Thus, the upskilling of medical practitioners, the development of prescription support software, and the engagement of a reference doctor or domain expert may help augment adherence to these guidelines [20, 21].

Our investigation attests that deviation from the recommendations does not significantly impact patient outcomes in terms of mortality and morbidity (*p* values of 0.698 and 0.923, respectively) [22]. These findings may be attributed to the advancement of surgical procedures, meticulous hemostasis implemented by surgical teams, and the evolution of anesthesia techniques, such as increased administration of norepinephrine over fluid resuscitation, potentially accounting for the variation in intraoperative bleeding volume. It has now been described that volume expansion with crystalloids can induce endothelial damage and increase the risk of reoperation, fistula risk, and suturing difficulties and overall might cause a dilutional coagulopathy leading to increased bleeding [23]. This is only a list of assumptions, as these indicators were not measured in our study. New prospective studies on a larger population with new variables measured are needed to reach a conclusion.

The predominant use of aspirin (64.1%) in the cohort, particularly for primary prevention, was not unexpected, albeit surprising. This practice remains widespread among general practitioners, despite recent studies advocating limited benefits of aspirin for primary prevention [24, 25]. The occurrence of bleeding events among patients receiving aspirin for primary prevention provides additional evidence advocating its discontinuation. The POISE-2 trial corroborates these findings by demonstrating that preoperative aspirin does not mitigate the risk of myocardial infarction but escalates the risk of bleeding events [26].

The coagulation status of patients administered DOACs has emerged as a topic of considerable interest [27–29]. Existing guidelines recommend discontinuation of DOAC therapy several days prior to surgery, even for low-risk procedures. However, the practicality and potential benefits of continuing DOACs during surgery, especially in emergencies, require further exploration. The inability to perform surgery on patients under DOAC-Therapy incurs substantial costs, inclusive of antagonist use or administration of high volumes of plasma or factor concentrates. Regrettably, our study did not include any patients undergoing surgery while still on DOACs.

The implementation of recommendations is multifaceted, particularly as medicine becomes increasingly personalized and the population ages. Ensuring accurate and comprehensible prescriptions for patients is crucial. The study suggests that strict adherence to guidelines may not be compulsory, allowing therapeutic education to concentrate on augmenting patient comprehension of their treatment. Providing clearer instructions to patients, such as specific dates for discontinuing medications, can improve adherence and reassure patients [30, 31]. Our analysis focuses on the “strict” part of therapeutic compliance. An adjustment of a few days (allowing simplification) on stopping treatment to increase compliance with the prescription may be a solution (for example in the case of polymedication with an antihypertensive or an antidiabetic with stopping all treatment on a specific day). Simple rules that are correctly applied are perhaps better than complex rules that are not correctly applied.

The study did not thoroughly investigate the resumption of treatment in hospital wards, which often relies on individual practices and can vary widely. This is a real confounding factor because the diversity and variety of these practices can lead to real differences in care and therefore differences in the primary outcome measured in this study. Enhancements in care are imperative to ensure a seamless transition and patient education during this period. Continuation of treatment can help prevent relapses, while early relapses can expedite patient discharge.

Our study has a number of weaknesses, the main one is that we did not study the resumption of post-operative treatment. The resumption of these treatments has a major influence on the risk of bleeding or thrombo-embolic events.

The number of patients included does not correspond to the previously calculated number of patients to be included. This is due to a faulty meter reading when patients were included. This is due to a faulty meter reading when patients were included. The inclusion record was in paper format, allowing the inclusion number to be matched with the identity of the papers so that the anonymity of the papers included could be respected during the statistical analysis. Unfortunately, one page was omitted, and 15 inclusions were missed.

There is no information on confounding factors in patient management. The correct administration of tranexamic acid, temperature management, or management of hypotension was left to the practitioner in charge of the patient, and the strict comparability of the 2 groups (compliance or non-compliance with recommendations) was not measured.

## Conclusions

The decision-making process for cessation of antithrombotic therapy often presents a challenge for anesthetists during preoperative consultations. This single-center study in an academic hospital found no effect of guideline non-adherence compared to adherence guidelines on 1-month survival. However, this absence of difference must be interpreted in the context of a probable lack of statistical power, a heterogeneous patient population with all specialties represented, and a mix of emergency and scheduled surgery patients. As the future of perioperative medicine is likely to lie in personalization of care, coupled with a rise in complex cases, there is a probable need for the development of prescription support tools for anesthetists.

### Supplementary Information

**Additional file 1.** STROBE Statement—checklist of items that should be included in reports of observational studies 

## Data Availability

The datasets during and/or analyzed during the current study are available from the corresponding author on reasonable request.
