# Supplementary material for: Influence of compliance to antithrombotic agents on perioperative morbidity and mortality
Source: J Anesth Analg Crit Care. 2023 Oct 18;3:38. doi: 10.1186/s44158-023-00123-5 (PMC10583434; doi:10.1186/s44158-023-00123-5)
Supplement: Supplementary file 1 — Additional file 1. STROBE Statement—checklist of items that should be included in reports of observational studies [file 44158_2023_123_MOESM1_ESM.docx]

**Annexes :**

**CONSORT 2010 Flow Diagram**

Assessed for eligibility (n= 1063)

## Enrollment

Excluded (n= 474)

  Included in interventional studies (n= 17)

  Declined to participate (n= 53)

  Organisational reason (n= 124)

  Pregnant women, children, unable to consent (n= 96)

  Neuroaxial anesthesia (n= 84)

  Vital emergencies (n = 100)

Alive (n = 560) Dead (n = 29)

  Haemorrhagic event (n= 28)   Haemorrhagic cause (n= 8)

  Ischemic event (n= 30)   Ischemic cause (n= 3)

  Without any event (n= 502)   Other cause (n= 18)

*Revision surgery (n= 91)*

Drugs Treatment modification Indications

  Acetylsalicylic acid (n = 378)   Non-stop (n = 310)  Primary prevention (n= 174 )

  Enoxaparin (n= 46)   Stop without bridge (n= 246)

  Acenocoumarol (n= 38)   Stop with Bridge (n= 33 )

  Clopidogrel (n= 24)

  Apixaban (n= 24)

  Rivaroxaban (n= 25)

Included (n= 589)

## Day of the

## surgery

## One month follow-up

Guideline adherence

  Adherence (n= 366)

  Non-adherence(n= 223)

**Figure 1 : Consort flow diagram**

## Anesthesiology consultation

**Figure 2 bleeding volume and length of stay regarding adherence to guidelines.**

*
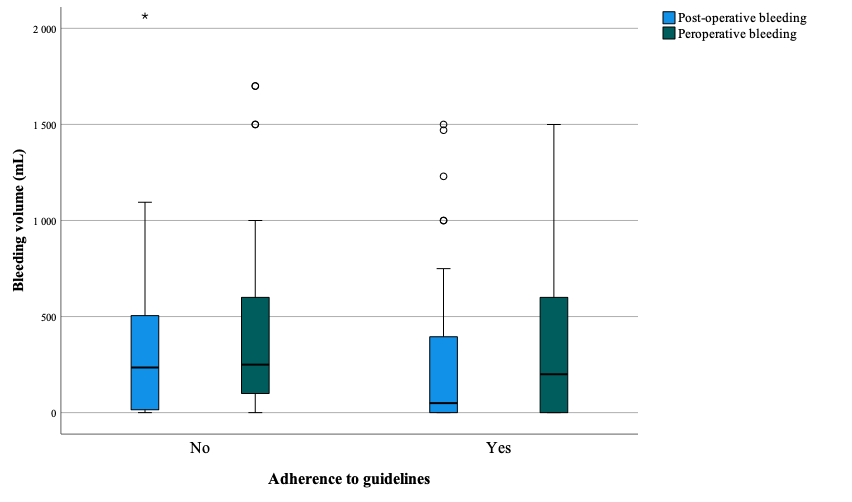
*

*Figure 2a: bleeding volume regarding adherence to guidelines (p-value = 0,460 for peroperative bleeding and 0,087 for post-operative bleeding)*

*
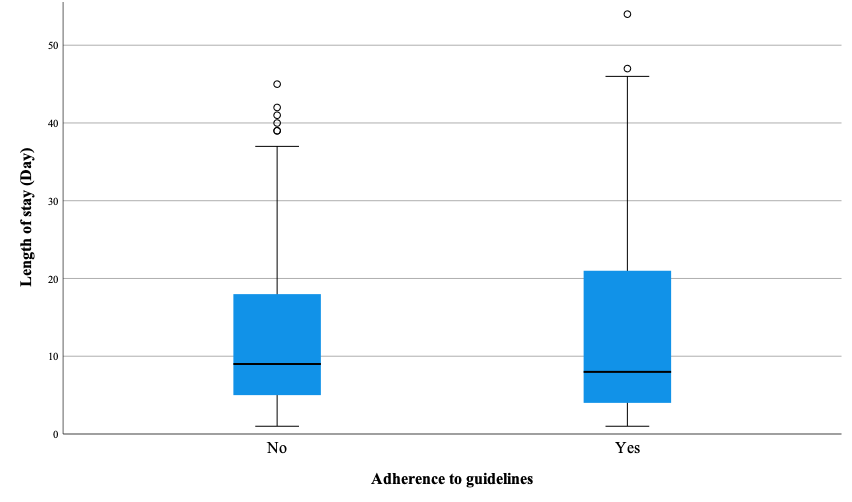
*

*Figure 2b: length of stay regarding adherence of guidelines (P-value = 0,339)*

|  | **Guideline’s adherence group** | | **Guidelines non-adherence group** | | P-Value |
| --- | --- | --- | --- | --- | --- |
|  | Mean | SD | Mean | SD |  |
| BMI (kg/m^2^) | 27,36 | 5,96 | 29,69 | 32,91 | 0,13 |
| Weight (kg) | 79,11 | 18,87 | 78,03 | 17,77 | 0,25 |
| Height (cm) | 169,50 | 9,73 | 167,63 | 12,85 | 0,99 |
| Age (years) | 78,06 | 152,30 | 81,33 | 144,85 | 0,02* |
| APTT (Sec) | 25,73 | 5,64 | 25,67 | 9,87 | 0,64 |
| PT (%) | 89,50 | 26,51 | 91,98 | 25,01 | 0,23 |
| INR | 1,23 | 1,55 | 1,10 | 0,31 | 0,05 |
| Hb (g/dL) | 12,69 | 2,42 | 12,53 | 2,30 | 0,68 |
| Platelets (/mm^3^) | 311 567,68 | 94 745,81 | 251 420,48 | 112 501,76 | 0,18 |
| GFR (mL/min/1.73m^2^) | 78,14 | 29,41 | 68,78 | 31,09 | 0,15 |

*BMI:* Body mass index

*APTT: pre-operative activated partial thromboplastin time*

*PT: pre-operative prothrombin time*

*INR: pre-operative international normalized ratio*

*Hb: haemoglobin*

*GFR: glomerular filtration rate*

**Table 1: demographic and preoperative biologic data (n= 589)**

|  | **Adherence to guidelines** | | | | **Justification** | | **Total** | |
| --- | --- | --- | --- | --- | --- | --- | --- | --- |
|  | No | | Yes | | sclerosis  of arterial  system and  left heart | venous  system and right heart |  | |
|  | N | % | N | % |  |  | N | % |
| ***Total*** | ***223*** | ***100,00%*** | ***366*** | ***100,00%*** | ***356*** | ***233*** | ***589*** | ***100,00%*** |
| No treatment | 0 | 0,00% | 26 | 7,10% | 0 | 26 | 26 | 4,40% |
| Acenocoumarol | 18 | 8,10% | 20 | 5,50% | 35 | 3 | 38 | 6,50% |
| Acetylsalicylic acid | 150 | 67,30% | 228 | 62,30% | 186 | 192 | 378 | 64,20% |
| Acetylsalicylic acid + dipyridamole | 0 | 0,00% | 1 | 0,30% | 0 | 1 | 1 | 0,20% |
| Apixaban | 11 | 4,90% | 13 | 3,60% | 24 | 0 | 24 | 4,10% |
| Clopidogrel | 11 | 4,90% | 13 | 3,60% | 24 | 0 | 24 | 4,10% |
| Dabigatran | 3 | 1,30% | 5 | 1,40% | 8 | 0 | 8 | 1,40% |
| Edoxaban | 1 | 0,40% | 3 | 0,80% | 4 | 0 | 4 | 0,70% |
| Enoxaparin | 13 | 5,80% | 33 | 9,00% | 36 | 10 | 46 | 7,80% |
| Unfractionated heparin | 1 | 0,40% | 3 | 0,80% | 4 | 0 | 4 | 0,70% |
| Nadroparin | 1 | 0,40% | 1 | 0,30% | 2 | 0 | 2 | 0,30% |
| Rivaroxaban | 8 | 3,60% | 17 | 4,60% | 25 | 0 | 25 | 4,20% |
| Tinzaparin | 6 | 2,70% | 3 | 0,80% | 8 | 1 | 9 | 1,50% |

**Table 2: Antithrombotic medication prescription distribution**

*Sclerosis of arterial system and left heart: Cerebral or coronary infarction, carotid atheroma*

*Venous system and right heart: Deep vein thrombosis or pulmonary embolism*

*No treatment : The patient was supposed to take a treatment long time before the surgery but has stopped on his own long time before also. The patient's medical file stated that the patient should take aspirin as primary prevention and the patient chose of his own accord to stop the treatment long before surgery. In this case, there was no change in the patient not taking the treatment.*

|  | **Adherence to guidelines** | | | | **Dead** | **Alive**  **with consequences** | **Alive**  **without consequences** | **Total** | |
| --- | --- | --- | --- | --- | --- | --- | --- | --- | --- |
|  |  |  |  |  |  |  |  | (Amongst  specialities) | |
|  | No | | Yes | |  |  |  |  |  |
|  | *N* | *%* | *N* | *%* | *N* | *N* | *N* | *N* | *%* |
| ***Total*** | ***223*** | ***100,00%*** | ***366*** | ***100,00%*** | ***29*** | ***58*** | ***502*** | ***589*** | ***100,00%*** |
| No identification | 0 | 0,00% | 7 | 1,90% | 0 | 0 | 7 | 7 | 1,20% |
| Anaesthesia | 0 | 0,00% | 2 | 0,50% | 0 | 0 | 2 | 2 | 0,30% |
| Digestive | 64 | 28,70% | 99 | 27,00% | 11 | 13 | 139 | 163 | 27,70% |
| Gynaecology | 5 | 2,20% | 11 | 3,00% | 2 | 2 | 12 | 16 | 2,70% |
| Neurosurgery | 13 | 5,80% | 54 | 14,80% | 1 | 10 | 56 | 67 | 11,40% |
| Obstetrics | 0 | 0,00% | 4 | 1,10% | 0 | 0 | 4 | 4 | 0,70% |
| Ear-Nose-Throat | 2 | 0,90% | 1 | 0,30% | 0 | 0 | 3 | 3 | 0,50% |
| Orthopaedics | 40 | 17,90% | 81 | 22,10% | 4 | 8 | 109 | 121 | 20,50% |
| Plastic surgery | 9 | 4,00% | 11 | 3,00% | 2 | 2 | 16 | 20 | 3,40% |
| Thoracic | 24 | 10,80% | 14 | 3,80% | 1 | 5 | 32 | 38 | 6,50% |
| Transplantation | 8 | 3,60% | 13 | 3,60% | 0 | 3 | 18 | 21 | 3,60% |
| Urology | 12 | 5,40% | 16 | 4,40% | 0 | 1 | 27 | 28 | 4,80% |
| Vascular | 46 | 20,60% | 53 | 14,50% | 8 | 14 | 77 | 99 | 16,80% |

**Table 3: Surgical speciality repartition regarding adherence to guidelines**

|  | Adherence to guidelines | | Total | |
| --- | --- | --- | --- | --- |
|  | No | Yes |  |  |
| Dead (within 30 days) | 12 | 17 | 29 | 5% |
| Alive with consequences | 22 | 36 | 58 | 10% |
| Alive without consequences | 189 | 313 | 502 | 85% |
| Total | 223 | 366 | 589 |  |
|  | 37,90% | 62,10% | 100% |  |

**Table 4: Adherence to guidelines and consequences on mortality or morbidity.**
